# Monitoring Various Bioactivities at the Molecular, Cellular, Tissue, and Organism Levels via Biological Lasers

**DOI:** 10.3390/s22093149

**Published:** 2022-04-20

**Authors:** Hongrui Shan, Hailang Dai, Xianfeng Chen

**Affiliations:** 1 State Key Laboratory of Advanced Optical Communication Systems and Networks, School of Physics and Astronomy, Shanghai Jiao Tong University, Shanghai 200240, China; hr.shan@sjtu.edu.cn (H.S.); hailangdai@sjtu.edu.cn (H.D.); 2 Collaborative Innovation Center of Light Manipulations and Applications, Shandong Normal University, Jinan 250358, China

**Keywords:** biolaser, microlaser, sensing of life, whispering-gallery mode

## Abstract

The laser is considered one of the greatest inventions of the 20th century. Biolasers employ high signal-to-noise ratio lasing emission rather than regular fluorescence as the sensing signal, directional out-coupling of lasing and excellent biocompatibility. Meanwhile, biolasers can also be micro-sized or smaller lasers with embedded/integrated biological materials. This article presents the progress in biolasers, focusing on the work done over the past years, including the molecular, cellular, tissue, and organism levels. Furthermore, biolasers have been utilized and explored for broad applications in biosensing, labeling, tracking, bioimaging, and biomedical development due to a number of unique advantages. Finally, we provide the possible directions of biolasers and their applications in the future.

## 1. Introduction

An embryo in its first stage of life undergoes an extraordinary transformation from a single cell to a dozens of centimeters long fetus with immune, digestive, and nervous systems, limbs and toes, etc. Tissue engineering involves myriad cellular and molecular events of complexity. Therefore, studying the various bioactivities at the molecular, cellular, tissue, and even living organism levels is very important to life science. Furthermore, the research provides a way to comprehend life’s nature and meaning. The most recent review and perspective papers on kinds of methods were published a decade ago. The specific fluorescence of a molecular trajectory is tracked by kinds of super-resolution methods from the fluorescence [1,2,3,4,5,6], material [7,8,9,10,11], and nanotechnology fields [12,13,14,15,16,17,18,19]. As we know, a large number of molecules comprise a cell, and various functional cells construct a tissue or organ, and there are many methods of researching cell bioactivities and tissue functions. A tremendous amount of research has been undertaken, with a significant number of papers published [20,21,22,23,24,25,26,27,28,29]. Compared to fluorescence, material, and nanotechnology, laser emission is an advanced method to detect various bioactivities at any life level. Biological lasers have many advantageous points in applications. The strong intensity of the laser beam leads to a high signal-to-noise ratio. The directional out-coupling of lasing is easily detected and monitored, and the perfect and high-sensitivity optical feedback mechanism senses small changes in biological processes in real time. Moreover, the narrow linewidth of the lasing beam leads to spectrally multiplexed detection, imaging, and tracking.

**Figure 1:**
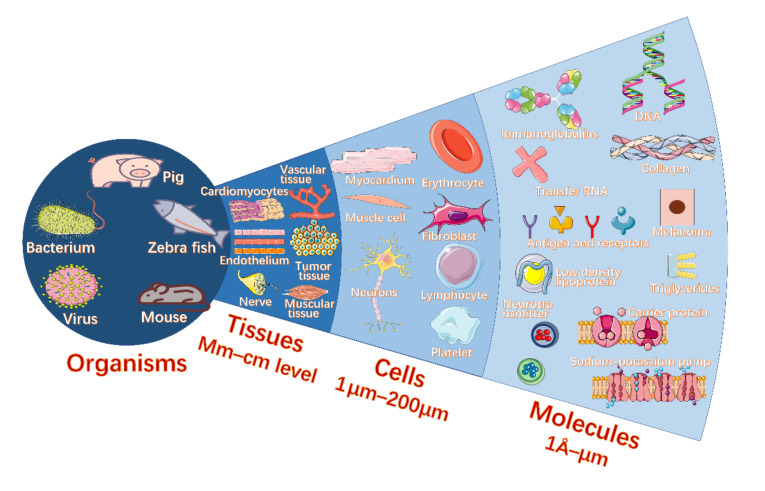
The overview of biolasers in different life levels, including organisms, tissues, cells, and molecules.

Owing to the strengths of biolasers, kinds of biolasers have been utilized to detect or monitor various bioactivities at the molecular [30,31,32,33,34,35,36,37,38,39,40,41,42,43,44,45,46,47,48,49,50,51,52,53,54,55,56], cellular [57,58,59,60,61,62,63,64,65,66,67,68,69,70,71,72,73,74,75], tissue [76,77,78,79,80,81,82,83,84,85,86,87,88], and organism levels [89,90,91,92,93,94,95,96,97], Recently, their applications in cell tracking, labeling/probes, implantable devices, and cell/tissue imaging have started to emerge. However, molecular, cell, and tissue functions should be independently distinguished in the complex biological field, and all of the previous reviews have not separated and described various bioactivities at the molecular, cellular, tissue, and organism levels with kinds of biological lasers. In this review, we discuss distinct biolasers and their potential biological and biomedical applications at all levels, as shown in Figure 1. In the end, we present an outlook on the possible challenges and future directions of biolasers.

## 2. Biosensing with Biolasers

### 2.1. Environment and Molecular Detection with Biolasers

Recently, the emerging technology of biolasers has offered highly sensitive ways to detect biomarkers contained in biological environments [33,34,36,39,41,42,43,51,56,97], such as pH, temperature, bio-electrostatics, humidity, and osmotic pressure, as well as some biological molecules such as proteins [30,32,37,38,46,47,50,52,53,54,55] and DNA [31,40,44,45,48,49]. In this aspect, the laser is an important tool in biological sensing due to its famous properties of perfect directionality, high intensity, and monochromatic emission. In the following parts, we present a series of outstanding achievements in the biosensor field.

#### 2.1.1. Biological Environment Detection

**Figure 2:**
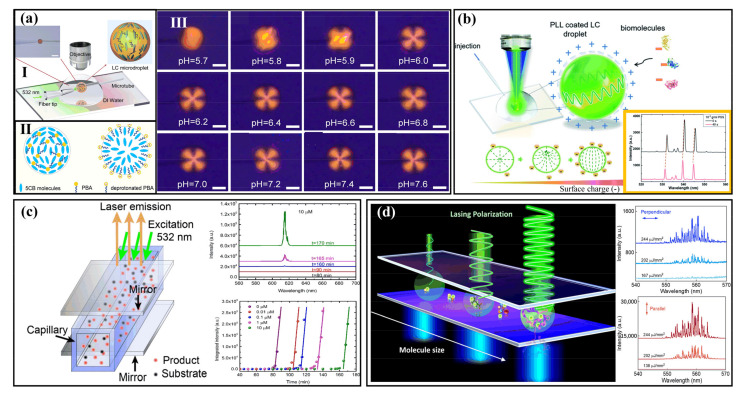
Environment and molecular detection with biolasers. (**a**) Ⅰ: Schematic diagram of liquid crystal (LC) microlaser setup. Ⅱ: Schematic diagram of structural change of microdroplets based on PBA-doped 5CB with different pH values (5.7, left; 6.0, right). Ⅲ: Polarized optical microscope (POM) figures of the microdroplets based on PBA-doped 5CB in PBS solution with distinct pH values. Scale bars 20 μm. (Adapted with permission from [34]. Copyright 2017 Elsevier B.V.) (**b**) The fixed LC C6-doped microdroplet was pumped with a laser. The microdroplets were coated with PLL solution to create a surface with a positive charge, which attracts molecules with negative charges. Inset marked in orange: lasing spectra collected before and after adding PSS solution to the LC microdroplet. (Adapted with permission from [43]. Copyright 2020 The Royal Society of Chemistry.) (**c**) Lasing cavity frame of the optofluidic catalytic laser, emission spectra, and threshold times of the optofluidic catalytic laser with different S^2−^ concentrations. (Adapted with permission from [30]. Copyright 2017 Elsevier B.V.) (**d**) Schematic diagram of FP microlaser. Molecules are sandwiched with an FP cavity. Spectrum figures show the difference between perpendicular and parallel polarized lasing of fluorescein binding with glycine. (Adapted with permission from [51]. Copyright 2020 American Chemical Society.)

Over the last couple of decades, many works about the detection of biological environments have been performed. For example, in pH sensing, Watanabe et al. utilized the intensity of lasering emissions based on a photonic crystal nanolaser fabricated with GaInAsP which is controlled by the change in the redox potential to identify the pH of a solution [35]. In 2017, a pH optical sensor based on random lasing was developed, and it showed pH sensitivity 200-fold higher than that of previous fluorescence sensors [38]. The high sensitivity makes the laser signal promising for biosensing applications. The detectable shifts in spectra are able to assume the role of an indication of the hydrogen ion concentration which is released from the enzymatic reaction. In the same period, Wang et al. reported a microdroplet-based pH sensor created by liquid crystals (LCs) and investigated the capacity of monitoring enzymatic reactions in penicillinase [34]. The combination of molecules and surfaces of LC droplets can alter the electromagnetic property and alignment of the LC droplet’s internal structure, resulting in distinct laser emissions with various wavelengths and modes. Figure 2a presents a pH sensor based on LC droplets doped with 4′-pentyl-biphenyl-4-carboxylic acid (PBA). Characterizing biosensing properties using changes in lasing spectra and modes is becoming more and more attractive. In this mechanism, the designed micrometer-sized microdroplets show transitions in configuration from bipolar to radial as the value of phosphate buffer solution (PBS) pH increases by 0.3, resulting in distinct signals in laser emission spectra.

In addition, electrostatics plays a critical role in a biological environment. As depicted in Figure 2b, in 2020, a bioelectrostatic-responsive microlaser fabricated using LC droplets was produced and employed for the identification of negatively charged biomolecules [43]. Similarly, the response of the charge and the molecular electrostatic variations on the surface of the LC microdroplet cause the lasing wavelength to shift in a dynamic range of 4 orders of magnitude. 

Because of the widespread adverse effects of sulfide ions on both environment and organism health, another biological environment study was carried out on the sensitive detection of sulfide ions based on an optofluidic catalytic laser [30]. As illustrated in Figure 2c, the gain medium for this lasing is highly fluorescent resorufin which is produced from a catalytic reaction. Utilizing sulfide ions inhibitors can slow down the catalytic reaction, which in turn leads to a time delay in the laser, thus providing sensing information. In consequence, a detection limit of 10 nM is obtained with a dynamic range of three orders of magnitude.

In addition, the biological environment contains many components. In 2020, a strategy was proposed to extract and amplify polarized lasing signals of small molecules [51]. By exploiting the coherence of the strong optical feedback and stimulated emission provided by the resonant cavity, the microrotation effect of the molecule can be amplified (Figure 2d). Via detection of molecular rotation correlation times by excitation and emission, small molecules such as glycine, L-leucine, L-glutathione, and insulin could be distinguished. As far as the authors know, this is the first time an optical approach that can recognize various small molecules has been reported. This method provides new insight into the sensing of the components of the biological environment.

#### 2.1.2. Protein and Protein Appendage Detection

Enzyme-linked immunosorbent assay (ELISA) is a potent approach for biomolecular detection. Traditional ELISA uses light intensity as the detection signal. Due to nonspecific binding, material autofluorescence, and excitation light leakage, it often encounters a large background, which affects its detection limit and dynamic range. Nowadays, biolasers are becoming a common trend in biological monitoring research. In 2014, Wu et al. demonstrated an optofluidic laser that allows ELISA to occur inside the laser cavity [56]. The laser onset time can serve as the sensing signal; it is inversely proportional to the enzyme concentration and thus can represent the analyte concentration. In this work, interleukin-6 was used, presenting a 1 fg/mL sensing limit and a dynamic range of 6 orders of magnitude, indicating a promising potential for biological sensors. Gong et al. utilized hollow optical fibers (HOFs) to provide a compact microcavity on the fiber, causing a uniformly distributed lateral lasing [32]. 1D DFOFL was intergraded by several microfluidic channels in order to realize chip-scale array monitoring of horseradish peroxidase (HRP) enzymes. By using wavelength and spatial multiplexing techniques in 2D microfluidic chips, wavelength tunability of hundreds of nanometers was developed for rapid, high-throughput, and multifunctional detection instruments. In 2018, Tan et al. performed improvements to the ELISA detection system. Experimenters directly microfabricated array microscale reaction wells on dielectric mirrors for sensing in ELISA. The resulting interleukin-6 detection limit of about 0.1 pg/mL was achieved [52].

**Figure 3:**
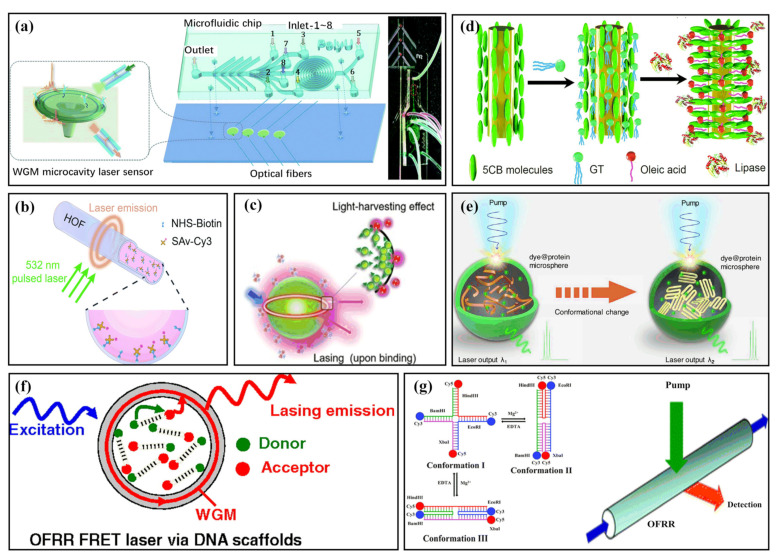
Protein, protein appendage, and DNA detection with biolasers. (**a**) Diagram of the WGM microlaser optofluidic biochip for ELISA and photo of the fabricated optofluidic chip. (Adapted with permission from [47]. Copyright 2020 The Royal Society of Chemistry.) (**b**) Schematic of the sequentially bioconjugated fiber optofluidic laser (FOFL). (Adapted with permission from [41]. Copyright 2021 The Royal Society of Chemistry.) (**c**) Illustration of interfacial FRET laser. (Adapted with permission from [42]. Copyright 2020 WILEY-VCH Verlag GmbH & Co. KGaA, Weinheim.) (**d**) Diagram of the molecular orientation of LC 5CB before and after the enzymatic reaction of lipase and glycerol trioleate. (Adapted with permission from [53]. Copyright 2020 The Royal Society of Chemistry.) (**e**) Illustration of the principle of biolasers based on silk protein conformation sensing before and after treatment with methanol. (Adapted with permission from [54]. Copyright 2021 American Chemical Society.) (**f**) Conceptual illustration of optofluidic ring resonator (OFRR) FRET lasing fabricated using DNA scaffolds. (Adapted with permission from [44]. Copyright 2010 Proceedings of the National Academy of Sciences of the United States of America.) (**g**) Left: The ion-dependent folding of the DNA Holliday junction with and without Mg^2+^. Right: Schematic of the OFRR bio-switchable lasing. (Adapted with permission from [49]. Copyright 2012 The Royal Society of Chemistry.)

With the development of 3D printing technologies, researchers have started to fabricate ELISA detection channels. As presented in Figure 3a, Ouyang et al. achieved rapid fabrication of high-Q disk whispering-gallery-mode (WGM) microcavities with the optical 3D printing technology which can be fabricated using maskless ultraviolet lithography [47]. This optofluidic chip widely used in ELISA is able to detect HRP-streptavidin by chromogenic reaction at a 0.3 ng mL^−1^ concentration. Additionally, the ability to differentiate the disease biomarker vascular endothelial growth factor (VEGF) at a very low concentration level of 17.8 fg mL^−1^ is more than 2 orders of magnitude higher than that of commercially available ELISA kits. Such a polymer biolaser-integrated optofluidic chip can offer a low-cost way to produce high-performance biochips for the sensitive diagnosis of multiple disease biomarkers. In 2021, a different approach based on a fiber optofluidic laser according to a sequential bioconjugation protocol for rapid testing without wash-out technologies for ELISA was proposed (Figure 3b). In this strategy, various chemicals were consecutively sucked into the miniaturized HOF to realize specific binding and residual rinsing with the capillary phenomenon. The wash-out-free, low-consumption, and high-integration approach can identify different concentrations of avidin from 10 pM to 100 pM. [41] Turbidimetric inhibition immunoassay (TIIA) is a similar method to ELISA and is also widely applied to biomarker sensing. Through the incorporation of the microlasers, immunoreaction occurs in the cavity, which significantly increases the signal via the amplification effect of the laser. A dynamic range of 5 orders of magnitude and an excellent detection limit of 0.18 ng/L were obtained [55].

Moreover, proteins, as the basic participants of vital life activities, control the final biological process of life activities. It is important to monitor the expression level of proteins in all stages of life activities. In 2016, milk containing rhodamine 6G was pumped by 532 nm laser pulses, resulting in a random lasing associated with the fat volume concentrations, showing that such a random laser technology is able to be applied to monitor the important fat volume concentration parameters [39]. As depicted in Figure 3c, in 2020, Yuan et al. employed Forster resonance energy transfer (FRET) to WGM microlasers. Various laser spectra can distinguish molecules of diverse absorption properties related to binding. Then, research about small fluorescent molecules and photosynthetic proteins was carried out [42]. This work not only proves the wide application potential of the microlaser outer cavity in molecular sensing applications, but also provides a new angle for cavity energy transfer in laser physics. Lipases perform a significant function in lipid metabolism. These enzymes are broadly found in almost all living things and control processes such as digestion, absorption, and metabolism. As shown in Figure 3d, authors developed a method of lipase concentration monitoring using a liquid crystal microfiber biolaser. Through the enzymatic reaction of lipase with triolein, a self-assembled monolayer of surfactant is formed at the LC/aqueous solution interface, and the laser can capture the surfactant and quantitatively characterize it by wavelength shift. With this method, lipase concentrations as low as 0.01 μg mL^−1^ can be discovered within 200 s [53]. However, it seems difficult to reuse the biosensor based on microfiber. The distinguishing of protein conformational changes has been the focus of much research because of the growing number of diseases linked to protein misfolding and aggregation, such as Huntington’s, Alzheimer’s, and Parkinson’s diseases. Biological microlasers are also applied to detect conformational changes in proteins. In 2021, microsphere biolasers fabricated using proteins were assembled by combining organic gain media with spherical silk fibroin with the use of emulsion-solvent evaporation (Figure 3e). The laser spectrum responded sensitively to the structural changes of the protein induced by the methanol vapor treatment [54]. This study provides an effective means for monitoring changes in protein conformation and demonstrates the relationship between a biological microstructure and its photon characteristics. In the same year, bionic liquid crystal droplets having self-assembling lipid monolayers were placed in a Fabry–Pérot chamber, and a topological transformation of the output vector beam triggered a subtle protein–liposome interaction. This work was proved by exploring the interaction between amyloid *β* (A*β*) and a lipid membrane, which can be regarded as a complex laser mode and polarization topology, which can offer a new idea for sensing [98]. Many other works about the detection of proteins, including fluorescent protein [50], human epidermal growth factor receptor 2 (ErbB2) protein [37], acetylcholinesterase [46], based on microlasers were carried out. These works offer important ideas for people to understand the role of proteins in life activities and provide monitoring methods for life activities at the protein level, which will be beneficial to the development of biomedical engineering.

#### 2.1.3. DNA Detection

DNA, which is important for all life forms, is a molecule consisting of two polynucleotide chains that are wound around to form a double helix, carrying genetic instructions about function, development, growth, and heredity for the vast majority of living organisms. Bioinspired optofluidic ring resonator (OFRR) FRET lasers that can be precisely controlled by DNA scaffolds were demonstrated, as displayed in Figure 3f. This kind of biolaser is a rising technology that cooperatively integrates microfluidics and a dye laser for miniaturized lasing sources possessing straightforward sample delivery and extremely small sample volumes [99,100,101,102,103,104]. Through DNA scaffolds, almost 100% energy transfer can be realized without considering the concentration of donor and acceptor. As a consequence, the laser can be obtained at a very low acceptor concentration, approximately 1000-fold lower than that of traditional optofluidic dye lasers [44]. DNA is one of the four important types of biomacromolecules which are essential in life processes. The normal activity of DNA determines genome stability to maintain life. However, changes to the DNA sequence may occur, namely base changes caused by biological processes such as mutation and cytosine methylation, which are of great significance in disease diagnosis and customization. In 2012, Lee et al. proposed an intracavity DNA melting analysis mechanism based on an optofluidic laser. The tiny signal change between the DNA with single-base mismatching and the target was optically amplified by the resonant laser, causing an orders of magnitude larger signal than fluorescence methods. The difference between two DNA sequences greater than 100 bases long was proved [40,48]. In addition, changing the structure of DNA can achieve the control of the biolasers. By controlling the Mg^2 +^ concentration in the solution, a DNA Holliday-based biofluidic laser was successfully manufactured (Figure 3g). The two laser gain media (Cy3 and Cy5) attach to the same DNA structure at the same time, and the invertible wavelength conversion can be realized by inducing DNA conformational changes (e.g., Mg^2+^) [45,49]. Another attractive property of DNA is that DNA is negatively charged because of the phosphate groups. The above-mentioned GaInAsP semiconductor photonic crystal nanolaser has the capacity to detect surface charge [35]. Once the device encounters the plasma and the charged DNA is adsorbed, a big wavelength difference Δλ exceeding 1 nm occurs. Hence, it also is used to identify DNA at very low concentrations [36]. Through various methods to distinguish error, DNA can play a key role in disease inspection, customized medicine, and basic life research [36,48,105,106,107]. In addition, DNA can be used to prepare biometric switching laser emission devices. A DNA-based self-switchable laser was proposed in which the laser uses a biological interface between the Fabry–Pérot microcavity in the nonlabeled DNA molecule and the dye-doped liquid crystal matrix. By using the DNA conformation and the change in the liquid crystal direction, laser emission switching between different wavelengths and strength is achieved, possessing a wide spectral range of reversibility and wavelength adjustability which can play an important role in biological monitoring [108].

### 2.2. Cellular Detection with Biolasers

#### 2.2.1. Extracellular Biolasers

Extracellular biolasers whose resonator is usually located outside the cell are commonly fabricated with a Fabry–Pérot (FP) cavity with two highly reflective mirrors [58,59,68,73,76,109,110,111].

**Figure 4:**
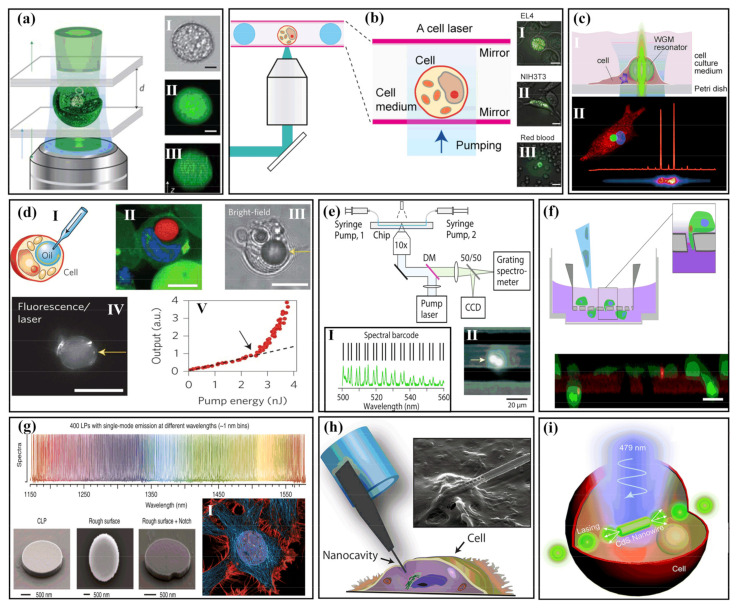
Cellular detection with biolaser. (**a**) Diagrams of the single-cell lasers. A living eGFP-expressing cell is captured by an FP cavity with two distributed Bragg reflectors. Ⅰ–Ⅲ: Figures of a single cell outside the cavity under the microscope. (Adapted with permission from [68]. Copyright 2011 Nature Publishing Group.) (**b**) Cells are injected between two highly reflective mirrors and then sink to the surface of the bottom mirror. The cells are irradiated in such a way that the whole cell or a series of cells is excited. Laser mode hyperspectral figures of various kinds of cells. Ⅰ, Ⅱ, Ⅲ: Superposition of bright field picture and laser picture (Adapted with permission from [59]. Copyright 2015 Optical Society of America.) (**c**) Ⅰ: Lasing based on live cells that internalize a microcavity. Ⅱ: Cytoplasm (red), nuclei (blue), and internalized microsphere resonators (green) of macrophages from cellular laser spectroscopy and confocal laser scanning microscopy (CLSM) information. (Adapted with permission from [64]. Copyright 2015 American Chemical Society.) (**d**) Ⅰ: Pictures of oil microinjection in the cytoplasm. Ⅱ, Ⅲ, Ⅳ: Cell pictures in the experiment. Ⅴ: Plot of droplet output light intensity versus pump pulse energy, presenting obvious lasing threshold (marked by red arrow). (Adapted with permission from [61]. Copyright 2015 Nature Publishing Group.) (**e**) Schematic of the microfluidic laser optical setup. Ⅰ: The wavelength series of WGM peaks recognized from the fluorescence subtraction spectra create the “spectral barcode” of cells. Ⅱ: Diagram captured by CCD of a cell (marked by yellow arrow) with two beads. (Adapted with permission from [71]. Copyright 2017 The Royal Society of Chemistry.) (**f**) Sketch (top) and experiment picture (below) of the movement of cells that ingested nanodisk microcavities through the microporous membrane. (Adapted from [66].) (**g**) Combination map of 400 single-mode emission laser microcavities of distinct wavelengths. Ⅰ: HeLa cells, defect-free semiconductor laser particles (CLPs), and surface-introduced defects give omnidirectional emission. (Adapted from [57].) (**h**) Photonic nanoprobe design and single-cell interrogation. (Adapted with permission from [69]. Copyright 2013 American Chemical Society.) (**i**) Graph of CdS NW lasing based on a cell. (Adapted with permission from [70]. Copyright 2018 The Royal Society of Chemistry.)

In 2011, two cooperation groups in the USA and Scotland, led by Seok Hyun Yun and Malte Gather, respectively, demonstrated the first extracellular biolaser with green fluorescent protein (GEP), as indicated in Figure 4a. By pumping with nanojoule and nanosecond pulses, the microlaser based on a transfected GEP cell produces high, directional, and narrowband laser emission. Compared with the surrounding media, the cytoplasm has a higher refractive index; the cell was able to act as a convex lens in the FP cavity which can be used to inhibit the extracellular laser. In addition, the threshold was only 14 nJ, which was much lower than the cell damage threshold [68]. In addition, to employ the cell as a gain medium, a gain medium based on dye can be injected extracellularly into the FP cavity. In 2015, Humar et al. demonstrated a more flexible experimental design in which the gain medium can exist everywhere in the FP cavity (Figure 4b). The laser resonant modes were determined by the shape and internal components of the cell, which possessed the ability to detect osmotic pressure [59]. Aside from gene transfection, synthetic fluorescent molecules can also be used to realize intracellular lasers. 5-Chloromethylfluorescein diacetate (CMFDA) was applied to the first cell laser demonstration. It only takes less than an hour to introduce synthetic dyes into normal living cells, which is much faster than instantaneous transfection that can provide sufficient fluorescent proteins for laser emissions [58].

The above three works have difficulty in achieving high-throughput sensors. Cell lasing analysis based on an integrated array platform is vital for high-efficiency sensing. In 2017, the Fan group proposed a microwell array with an FP cavity for cell-based laser research. Various cells were trapped in the microwells, and then the emission occurred under the pumping laser. The multichannel lasing signals could provide a tool for cell tagging, trapping, and abnormal identification [73]. Furthermore, it does not need a high-quality FP cavity to support cellular lasing; it only needs dyed cells with low-quality factor resonators made up of a disposable poly(methyl methacrylate) (PMMA) cell counting plate. The simple method without conventional high-reflectivity optical cavities allows a rapid expansion of cellular lasers [58].

#### 2.2.2. Intracellular Biolasers

##### Intracellular Microlaser Based on Microspheres and Microdisks

Intracellular biolasers refer to the resonators with gain medium internalized by cells, in which internalization is usually achieved by phagocytosis. Whispering-gallery-mode (WGM) microlasers are popular in intracellular applications and have many appealing features, including small size, high Q-factor, and low lasing threshold [57,60,61,62,63,64,66,67,71,72,73,74,89,112,113,114,115].

To obtain a high Q-factor, which is significant for sensing applications, WGM resonators need near-spherical, smooth surfaces with small scattering losses. The first demonstration of an intracellular spherical biolaser was realized by Hummar et al. with Nile-red-dyed oil microdroplets acting as soft WGM cavities in cells, as exhibited in Figure 4d. These authors injected the oil into the cells using a microsyringe attached to a glass microtube with an outer diameter of 1 μm. Due to the immiscibility of the oil droplets and the pressure of the cytoplasm, the droplets can keep good shapes. The droplets were first introduced into the cells, and the free space exciting approach of the oil immersion objective was used to excite the WGMs and collect the signal. Fortunately, there are cells that naturally contain lipid droplets, such as adipocytes. With the development of various fields, many similar cavities have appeared [116,117]. Following mixing with lipophilic fluorescent dyestuff and excitation by a nanosecond laser, an adipocyte containing a single lipid droplet realized WGM lasing. Aside from soft cavities, solid cavities are also popular due to their stability and usability [118,119,120]. A non-deformable WGM cavity can be produced using solid microspheres, such as polystyrene (PS) microspheres. PS microspheres are easily internalized by cells. Researchers achieved different WGM lasers with multiple positions of gain material, distributed on the inside, outside, and surface of the beads. Moreover, distinct PS microspheres with various diameters could create a set of output spectra that can operate as a fingerprint identification code for cell tagging, tracking, and sensing [61].

In 2015, the Gather group realized long-time single-cell tagging and tracking in experiments, which can be utilized for diverse cell types. Cells embedded with microsphere cavities can survive for several weeks in normal circumstances. The method was applied to identify and track different primary macrophage cells, as shown in Figure 4c [64]. In order to obtain a more precise and wider range identification ability, barcoding based on WGM is attractive for its noninvasive and repeated action at high speed. Intracellular microspheres with diameters from 5 to 12 μm created barcodes on basis of lasing spectra, as shown in Figure 4e. Microlasers in different cells have distinct barcodes. By comparing them to the barcodes in a database, identification with high accuracy is achieved [71]. In 2017, Schubert et al. took advantage of cell tagging and tracking technology based on WGM lasers to realize the long-term analysis of mitosis in real time. The authors performed tracking experiments on mitotic 3T3 fibroblasts. Over the course of the experiment, the cell underwent three cycles of cell division. The cell completed division, and the two daughter cells each received a microcavity from the mother cell and could create a lasing signal for tracking separately, which carries major implications for the study of the life activity of cells [72]. There are many other materials for WGM cavities [116,117,118,119,120], such as high-refractive-index microsphere (*n* = 1.9) [61], biocompatible protein microspheres [74], and organic microspheres [62]. For instance, in 2017, Ta et al. designed biolasers that are assembled by bovine serum albumin (BSA) protein and biological polysaccharide extracted from terrestrial plants. This approach of microsphere fabrication based on biomaterials allows long-term implantable biosensing devices [74].

In addition to microsphere WGM cavities, microdisks [57,66,89] are also famous for WGM cavities. In 2018, Fikouras et al. designed a process of high-throughput manufacture and intracellular integration of nanodisk lasing (Figure 4f). Utilizing the huge gain and high refractive index of quantum wells fabricated with GaInP/AlGaInP, a lasing threshold 500 times smaller than the energy usually found in two-photon microscopy with the threshold of 0.13 pJ was achieved. Long-term and uninterrupted lasing excitation on nanodisks in human macrophages, NIH 3T3s, primary mouse neurons, and primary human T cells showed that the life activity of a single cell can be monitored in real time [66]. Moreover, as shown in Figure 4g, in 2021, Toropov et al. studied the stability of laser emissions from microcavity disks in HeLa cells for cell tracking applications. Combined with barcode technology, the laser signal emitted by cells can be continuously tracked for up to 2 h, and the microcavity in the cell cytoplasm can be stably observed without losing the signal [57]. Apart from microsphere and micodisk cavities, microring, microbubble, microtube, and microgoblet WGM cavities have been demonstrated in biosensing.

##### Intracellular Biolasers Based on Nanowires

Nanowires and nanotubes are able to stably penetrate the cells, with potential applications for intracellular biodetection [36,40,41]. Compared to the internalized WGM cavity, most signals based on nanowires connected with fibers are easy to collect. A nanowire cavity with a tapered tip fiber could introduce light into living cells and is able to obtain lasing signals from subcellular regions at ultrahigh spatial resolution. A nanowire can deliver payloads into cells. For example, quantum dots (QDs) were attached to a nanowire tip. After the quantum dots were inserted into Hela cells, the functional nanowire released the QDs into the desired location within 1 min of ultraviolet laser illumination. A highly directed laser from a nanoprobe can selectively excite the QDs, which can perform ultrahigh-resolution and ultrahigh-contrast subcellular microoperation [65].

Long work time is necessary for cell sensing. Shambat et al. performed vitality studies of nanowires. Their experiment showed that the probe possessed minimal cytotoxicity to cells and the laser could be guided into cells and monitored for serval days, as shown in Figure 4h. In this process, the cells can undergo regular division. Additionally, a device for the detection of streptavidin represents a route to real-time biomarker and biomolecule detection within single cells [69]. Nanowires are able to be internalized like WGM cavities and act as individual microlasers in cells. A cadmium sulfide (CdS) nanowire microlaser was demonstrated that can be spontaneously internalized into cells and serve as an independent intracellular probe based on green laser emission whose peak linewidth is very narrow, only 0.5 nm (Figure 4i). Upon regulation of the cell culture medium of human umbilical vascular endothelial cells (HUVECs) with sodium chloride (NaCl) solution, HUVECs first contracted and then partially recovered [70]. During this progress, changes in cell volume led to variations in intracellular solute concentration and then resulted in a change in the refractive index of cell cytoplasm. By monitoring the lasing wavelength of the nanowire which is related to the surrounding refractive index, the cellular environmental changes could be observed in real time.

### 2.3. Tissue-Based Sensing with Biolasers

**Figure 5:**
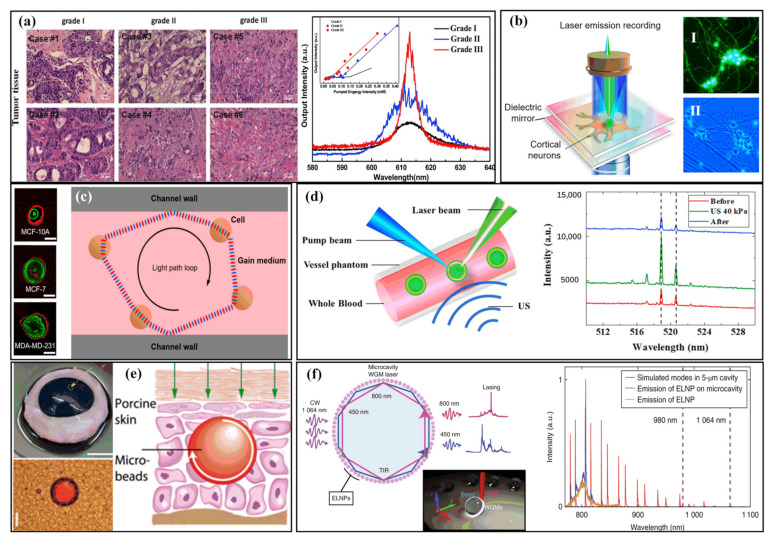
Tissue-based sensing with microlasers. (**a**) Microscopic pictures with HE dye and lasing spectra of human breast tumor tissues: malignancy grade I, malignancy grade II, and malignancy grade III. (Adapted from [83].) (**b**) Diagram of the laser path loop in a biofluidic random laser. The pink background indicates the gain medium in the solution. Ⅰ, Ⅱ, Ⅲ: Immunostaining of three kinds of cells. (Adapted with permission from [87]. Copyright 2019 American Chemical Society.) (**c**) Diagram of the configuration for neuron lasing, where neurons are trapped in an FP cavity composed of two highly reflective mirrors. Ⅰ: Fluorescence image of OGB-1-labeled primary rat cortical neurons cultured on a mirror. Ⅱ: Brightfield image of a neuron on a mirror. (Adapted with permission from [76]. Copyright 2020 American Chemical Society.) (**d**) Ⅰ: Position of injection of the bead microcavity dispersion. Ⅱ: A 40 μm diameter bead in blood. The beads are surrounded by red blood cells. Ⅲ: Principle of operation of a laser in skin tissue. (Adapted with permission from [77]. Copyright 2017 Optical Society of America.) (**e**) Ⅰ: Oil droplet controlled by ultrasound lasers in blood. Ⅱ: Laser spectroscopy of oil droplet laser mixing human whole blood and applying ultrasonic pressure before, during, and after loading into capillaries. (Adapted with permission from [84]. Copyright 2019 American Chemical Society.) (**f**) Ⅰ: Diagram of exciting and lasing in energy-looping nanoparticle (ELNP)-coated beads. Inset: Diagram presenting WGMs in a microsphere. Ⅱ: Simulated NIR spectrum of WGMs supported by a 5 µm polystyrene microsphere, overlaid on the experimental emission spectrum of ELNPs and ELNP-coated beads pumped near excitation. (Adapted from [88].)

Apart from cellular microlasers, lasing in tissues, which comprise a series of cells trapped in the complex extracellular matrix, is very attractive in real applications. Because it can create the actual biological environment in vivo. As a result, tissue lasers may lead to a wide range of applications in biological detection, disease recognition, and histological engineering. Due to the light scattering characteristics of tissues, it is difficult to ensure the quality transport of light in tissues. Hence, laser-speckle imaging is usually employed to monitor deep tissues [56]. However, it is the scattering property that leads to the prevalence of random lasers, as it is easy to achieve by combination with fluorescent dyes and all kinds of tissues, such as muscular tissue, nervous tissue, brain tissue, bone tissue, and some cancerous tissues [121,122,123,124,125]. Because random lasing is strongly linked to the structural features of random material, the emission signal is suitable for extracting structural information [81,82,83,87]. As shown in Figure 3a, in 2017, Wang et al. investigated the sensing ability of a random tissue laser embedded with a nanotextured organic dye applied to healthy and cancerous breast tissue. The findings showed more lasing signals in cancer tissues since there was more disordered scattering in cancer tissue. Therefore, the divergence of random tissue lasers with various malignant grades can be utilized for diagnosis [83]. Most of the traditional random lasers are used for solid tissues with dense scattering. In 2019, He et al. demonstrated a random laser cytometer in an optofluidic chip with dilute human breast normal and cancerous cells (Figure 5b). Light was scattered multiple times between microscale human cells and promoted random lasing. Through the power Fourier transformation of the lasing signal, cancerous cells in tissue mixtures can be diagnosed [87].

Due to the much greater scattering and absorption of tissues than cells, a greater light field strength is required to obtain the signal. An FP cavity is an excellent choice that can support a high-power standing-wave field. In this regard, Fan’s group has completed a series of studies. A scanning laser emission microscope for mapping laser emission in human tissue was proposed. Patients’ tissues labeled with particular dyes for various biomarkers were sandwiched in an FP cavity. Subsequently, an optical parametric oscillator (OPO) pulsed laser was guided into the cavity to build a laser-emission image that can show the deviation between cancer and normal lung tissues [79]. Similar lasers were proposed for many other tissues, such as bone tissue, blood tissue, muscle tissue, adipose tissue, and other functional tissues. As depicted in Figure 5c, by exciting and recording single neurons from neural networks, neuron activities can be recognized, which can help us understand neurological diseases and signaling [76].

Besides random lasers and FP lasers, implantable and biosafe WGM lasers are good choices for tissue microlaser detection [77,80,84,88]. For example, as shown in Figure 5d, several types of biocompatible microlasers assembled using dye-doped PS and polymer microspheres were embedded in the cornea and skin. Under the pumping of 475 nm and 5 ns pulsed lasers, the microlasers performed lasing of cornea tissues. Moreover, a single bead implanted by a tattoo machine at a depth of 100 μm below the skin surface was irradiated by a 532 nm 1 ns laser. There was a strong and narrow emission that possessed narrow linewidth (<0.2 nm) and a low threshold (<4 μJ) at the fluorescence peaks [77]. However, fixed-size microspheres can only support a certain range of refractive index environments. Microspheres with dynamic and adjustable lasing are of great significance in practical application. For this improvement, Li et al. proposed an ultrasonic modulated droplet laser based on WGM, as presented in Figure 5e. When the ultrasonic pressure exceeds a certain threshold, the laser power of the oil droplet can be increased 20 times. Dynamic lasing was achieved with various ultrasound frequencies and pressures, which can bring more options for biosensing [84]. Small cavities inevitably lead to large losses and require larger input powers to reach lasing thresholds. Therefore, most of the microlasers are realized by pulsed lasers with high peak power. For a wide application, the replacement of pulsed lasers with low-cost continuous-wave (CW) lasers is encouraged. In 2018, Fernandez-Bravo solved this problem with the energy-looping pumping method in Tm^3+^-doped upconverting nanoparticles (UCNPs) to realize CW upconverted lasing in independent 5 μm microcavities under the excitation energy of 14 kWcm^−2^ (Figure 5f). Coupling the energy-looping nanoparticles with the WGM of PS microspheres resulted in stable lasing for up to five hours at blue and near-infrared wavelengths. When the microcavities were immersed in fetal bovine serum, lasing was realized with CW excitation in the biologically transmissive second near-infrared window, which shows the ability of microlaser sensing with CW pumping [88]. The researchers demonstrated that optical microcavity probes entering intracellular microcavities can be perceived with a single cell for a long period of time over the depth of the optical transmission length, while they could also be perceived through the particular spectrum characteristics of the WGM, which are not subject to tissue scattering, absorption, and spontaneous fluorescence [126].

### 2.4. Monitoring the Life Activities of Living Organisms with Biolasers

**Figure 6:**
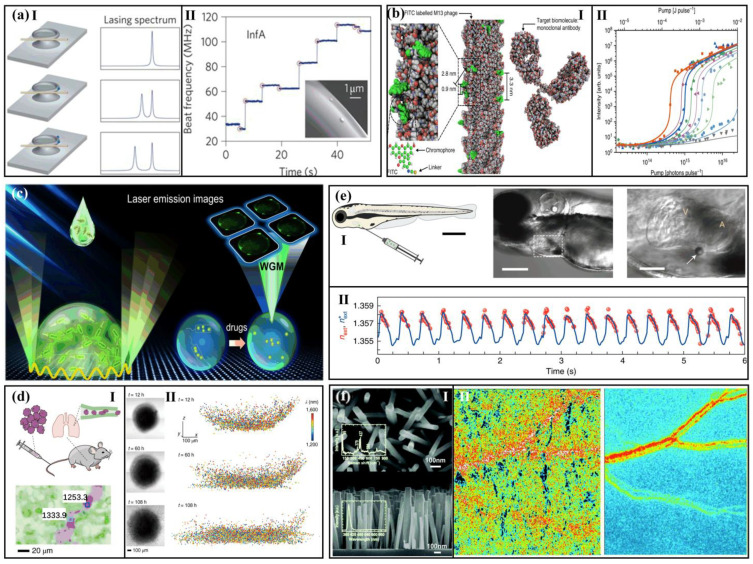
Monitoring the life activities of living organisms with biolasers. (**a**) Ⅰ: Diagrams presenting how the lasing spectra change as one and two nanoparticles (blue spheres) are connected to the microcavity. Ⅱ: Discrete variation in beat frequency with time when influenza A virions are bound to a WGM microlaser. Red circles represent single virus binding situations. Inset: Two InfA virions on the microlaser captured by scanning electron micrograph. (Adapted with permission from [91]. Copyright 2011, Nature Publishing Group.) (**b**) Ⅰ: Atomic structural model of the M13 phage laser probe covalently modified with fluorescein dye. Ⅱ: Threshold behavior based on virus-lasing probes with different fluorescein concentrations. (Adapted from [92].) (**c**) Diagram indicating the concept of the image-based E. coli biolaser microarray. (Adapted with permission from [93]. Copyright 2021 American Chemical Society.) (**d**) Ⅰ: Cells marked with laser particles (LPs) and fluorochrome were injected intravenously into a living mouse. Ⅱ: Optical communication of the tumor spheroid at different times (12 h, 60 h, and 108 h). Spatial distribution of LPs among tumors. Each point on the graph represents an LP, and its wavelength is color-coded. (Adapted with permission from [89]. Copyright 2019 Springer Nature.) (**e**) Ⅰ: Diagram of microlasers in living zebrafish embryos. Ⅱ: Two to four microlasers are inserted into three embryos, and contraction information is received from three microlasers in one embryo. (Adapted with permission from [96]. Copyright 2020 Springer Nature.) (**f**) Ⅰ: SEM images for the ZnO nanowires synthesized. Ⅱ: Comparison of mouse skin speckle pictures below (left) and above (right) the laser threshold. (Adapted with permission from [90]. Copyright 2019 The Royal Society of Chemistry.)

We have reviewed a series of biolaser sensors related to different life stages, including biological molecules, cells, and tissues. From the clinical application perspective, the ultimate goal of biosensing is living organism detection. Although they are the simplest biological system, viruses influence almost every organism [91,92]. As shown in Figure 6a, He et al. proposed a real-time microlaser based on WGM to detect influenza A virions in air. The WGM microlaser has a toroid-shaped construction prepared by erbium-doped silicon dioxide with a 20–40 μm diameter. In this method, a single virus binding event was translated into discrete frequency shifts of the heterodyne beat information which can be detected by a monitor [91]. Nevertheless, such a sensing device is unable to identify specific biomarkers. To achieve this goal, a biolaser fabricated with an M13 phage, which is a rod-like filamentous phage with a 7 nm diameter and a 900 nm length, for biosensing was introduced, as shown in Figure 6b. Making use of phage display, the M13 phage can be routinely programmed to bind diverse particular target biomolecules, resulting in a specific detection capability and showing a high sensitivity of less than 100 fmol mL^−1^ [92].

Representing another application in addition to virus sensing, bacteria are relatively widely distributed in the organisms of the world [93,94]. In 2021, to achieve high-throughput application, a microdroplet laser array encapsulated by Escherichia coli was printed on a high-refractive-index mirror, as illustrated in Figure 6c. With the pumping of 490 nm wavelength, a laser emission image that can serve as evidence of dynamic changes in living organisms was obtained [93]. More exciting results are emerging in larger organisms, such as zebrafish and mice [89,90,95,96]. Large-scale single-cell tracking was achieved based on WGM laser microdisks made of silica-coated semiconductors, which can support single-mode emission over 1170 nm to 1580 nm with sub-nanometer linewidths. Cells stained with dyes and loaded with microparticles were injected into the tail vein of a mouse to mimic a model of blood-vein micrometastases. Fifteen minutes later, the lung tissue was imaged, showing the ability of multiplexed tagging of a large number of cells even in scattering tissues. More details about the three-dimensional tracking of the tumor spheroid model are depicted in Figure 6d [89]. Recently, Gather and co-workers achieved organ monitoring with the aid of WGM microlasers, as shown in Figure 6e. Microsphere resonators with diameters between 10 and 20 µm were inserted in the heart, receiving a wavelength shift of 50 pm caused by the cardiac contraction, which allowed accurate monitoring of organ-scale contractility in live zebrafish with extraordinary repair and regeneration ability and mouse heart tissues. The microspheres were implanted using a micro-syringe into the outer wall of the atrium of the zebrafish. Under the optical pumping, lasing wavelength from microlasers displayed a redshift corresponding to the heartbeats [96].

Aside from WGM microlasers, random lasers were also successfully applied in mice, which was the first application of curvature-tunable random in vivo biological imaging, as demonstrated in Figure 6f. The device was assembled with disordered scatterers composed of ZnO nanowires, well-known piezoelectric materials, whose curvature could be modulated by bending the flexible polyethylene terephthalate (PET) substrate [90]. In this way, the spectral emissions of a random laser excited by such a tunable light source were constructed in in vivo mouse ear skin images with high speckle contrast, possessing a good ability for the study of the physiological phenomena of fast movement such as hemodynamics.

## 3. Outlook, Challenges, and Conclusions

Although multiple technological advances have been achieved in the past decades, as a rising and attractive technology, there are many directions that can be used and explored in the field of biolasers; meanwhile, biolasers are still facing challenges: (1) According to the results of previous studies, construction for high-throughput cell and tissue analysis will be interesting and important for biological research and biomedicine. The existing biolasers are based on a cell array in which individual cells reside in walls or flow through a microfluidic channel, resulting in an imaging limit at the cellular and tissue levels with biolasers, especially in time series analysis. (2) Rich spectral characteristics of the laser can be utilized, including narrow linewidth, mode profile, and polarization. Cell tagging and tracking via lasing micro/nanosize markers with multiple characteristics will become increasingly attractive. (3) In the detection of pH, temperature, local biomolecular concentration, local force/pressure, etc., lasers are highly sensitive to any environmental small changes. (4) Compared with conventional technologies such as X-ray testing, ultrasonic testing, computed tomography (CT), and magnetic resonance imaging (MRI), the biolaser can provide a way to realize super-resolution imaging achieving single-molecule biosensing, labeling, and tracking. Meanwhile, the biocompatibility of biolasers can protect the biological material from electromagnetic radiation. Therefore, biolasers can be used for cell monitoring purposes, and every individual cell can be uniquely observed.

Some properties are intrinsic to all biolasers, and others may be related to a particular laser cavity, biomaterial, or application. In the following, we list several challenges that we hope will be overcome by future research and development: (1) The cavity of the laser is in need of high pump intensity when the cavity has a relatively low Q-factor or low concentration of the gain medium, resulting in the damage of biological samples. Meanwhile, sophisticated and expensive equipment is also required, which limits the application of biolasers. Therefore, researchers of biolaser application hope that the smaller and inexpensive continuous-wave lasers can take place of the expensive pulsed pump in the future. (2) Due to the accumulative effect of laser output along the depth direction, information from the depth direction may be lost during laser imaging. Thus, the recovery of lost information in biolaser imaging would be a significant step in the biolaser development. (3) According to the theory of laser emission, a biolaser requires the fluorophore to reach a certain concentration to overcome the threshold with the help of external pumping. However, the low fluorophore concentrations of the molecules and cells may be missed due to being below the lasing threshold. (4). The intensity of a biolaser is weak, and the signal of lasing is absorbed by environmental materials. Moreover, the traditional methods employ perfect signal collection and processing systems. Therefore, biolasers are combined with traditional methods to explore new and interesting fields. Thus, the low labeling concentration or low density of molecules that can be successfully identified by enhancing the dynamic range would be another challenging yet significant topic in the biolaser application field.

## Data Availability

Not applicable.
